# The Impacts of the COVID-19 Pandemic on Therapy Utilization Among Racially/Ethnically and Socio-Economically Diverse Autistic Children

**DOI:** 10.1007/s10803-023-05905-y

**Published:** 2023-02-09

**Authors:** Cassin W. Gonzales, Jennifer R. Simonell, Mark H.C. Lai, Steven R. Lopez, Jonathan Tarbox

**Affiliations:** Department of Psychology, University of Southern California, Los Angeles, United States

**Keywords:** COVID-19, Autism spectrum disorder, Treatment access, Racial disparities, Socio-economic disparities, Multilevel modeling

## Abstract

**Purpose:** The purpose of current study was to evaluate change in hours of Applied Behavior Analysis (ABA) therapy utilization for autistic children during the year prior to the COVID-19 pandemic, the first three months of the pandemic (crisis phase), and the following 9 months of the pandemic (mitigation phase). Additionally, this study aimed to evaluate if change in therapy utilization differed based on child race, ethnicity, and primary payer of services. Finally, we aimed to identify potential mechanisms of ABA therapy disruption by interpreting findings using an extended version of Donabedian’s structure-process-outcome model. **Methods:** Retrospective clinical data on client demographics and therapy utilization (n = 283) were collected from ABA clinics in California and analyzed with four piecewise growth multi-level models. **Results:** We found that therapy utilization dropped during the first three months of the pandemic (-10.65 h/month; p < .001) and increased during the following 9 months (2.39 h/month; p < .001). Moderator analyses revelated that Asian, Non-Latinx and school-district funded children had significantly different trajectories of change in therapy utilization compared to white, non-Latinx participants and private insurance funded participants, respectively. **Conclusion:** Findings suggest that utilization of ABA therapy was disrupted for a full year following the onset of the COVID-19 pandemic and that child race/ethnicity and primary payer influenced the degree to which autistic children were impacted by service disruption. These findings have implications for autistic children who lost therapy access during key developmental periods and for the ABA care delivery system.

Global restrictions to in-person gathering brought on in March 2020 by the COVID-19 pandemic have significantly impacted children’s access to educational and healthcare services. The impact of COVID-19 restrictions is of particular concern for autistic children^1^ who are recommended to receive time-intensive and face-to-face therapeutic services through early and middle childhood (Elder et al., 2017; Magiata, 2012). Autism Spectrum Disorder (ASD) support services (e.g. applied behavior analysis, occupational therapy, psychological counseling) aim to help participants develop skills and overcome challenges in order to move towards adaptive and/or values-based functioning. Early research on the impacts of the COVID-19 pandemic suggest that autistic children and their families experienced heightened challenges during the pandemic that necessitated more support than was required prior (Bellomo et al., 2020). For example, compared to before the pandemic, autistic children experienced more frequent challenging behaviors and higher levels of distress (Colizzi et al., 2020; Nunez et al., 2021; Hosokawa et al., 2021). In addition, the onset of the pandemic was associated with higher levels of psychological distress and concern for the future among parents of autistic children (Corbett et al., 2021; Kalb et al., 2021; Neece 2020).

Access to ASD support services during the pandemic is of particular concern in the face of these additive needs experienced by autistic children and their families. Between 2020 and 2021, three large parent-report survey studies found that many autistic children had disruption to at least one ASD related service during the COVID-19 pandemic. Specifically, 36.8 − 77% of parents reported disruption to applied behavior analysis (ABA), 56.7 − 80% reported disruption to special education, 47 − 59.8% reported disruption to speech-language therapies, 46.8 − 65% reported disruption to occupational/physical therapy, and 42% reported disruption to all therapy services (Allison & Levac, 2022; Bhat, 2021; White et al., 2020).

While the studies reviewed here clearly demonstrate increased need and disrupted access to services among autistic children and their families, the existing research leaves at least three important questions unanswered. First, how did access to ABA services change month-to-month in the pandemic as service providers began implementing mitigation efforts? Second, did ABA service disruption during the pandemic differentially impact children of different races, ethnicities, and socio-economic statuses? Finally, what potential mechanisms within the ASD care delivery system mediated the pandemic’s impacts on therapy utilization among autistic children?

## Changes in Access Through Different Phases of the Pandemic

Changes in restrictions and mitigation efforts in the first year of the COVID-19 pandemic suggest that there are two distinct theoretical phases, the crisis phase and the mitigation phase, which followed a pre-pandemic period. In order to evaluate changes during the two pandemic phases, it is helpful to first characterize a year-long pre-pandemic phase as a point of comparison. Prior to the onset of the COVID-19 pandemic, it would be expected that therapy utilization would vary throughout a year due to progress on goals and seasonal breaks. Generally, a child would have the highest intensity of ABA therapy utilization at the beginning of treatment when they have the most need. As a child makes progress in therapy and reaches treatment goals, the intensity (i.e. number of hours) would decrease in order to minimize workload for the child (Board, 2014). Given this pattern, it is expected that a child’s ABA therapy utilization would decrease over a 12 month period. Additionally, seasonal events (e.g. summer break, winter holidays) would likely lead families to take periodic short breaks from therapy. Therefore, the pre-pandemic phase can be characterized by semi-consistent ABA therapy utilization with periodic disruptions and gradual reductions through time.

The crisis phase began with the declaration of the COVID-19 pandemic on March 11, 2020 (World Health Organization, 2020), followed by California issuing the first stay-at-home order in the US on March 19th, 2020 (Exec. Order No. N-33-20, 2020). By March 30th, 2020, all but one US school district closed for instruction and 100,000 US schools remained closed for at least 8 weeks (through May 2020; Zviedrite et al., 2021). Thus, the crisis phase of the pandemic may be considered to have lasted roughly between March 2020 and May 2020 and was characterized by rapid shut downs and preparation for renewing access to therapeutic services.

Early in the crisis phase, changes in health delivery policy laid the groundwork for mitigation efforts (e.g., Medicaid and Medicare approved funding for health services delivered through tele-health; CMMS, 2020). By June 2020, large scale efforts to continue delivery of therapeutic services in the pandemic went into effect, signifying the beginning of what could be considered the “mitigation phase”. This transition from the crisis phase to mitigation phase is primarily evidenced by the finding that, by the end of June 2020, there was a 10-fold increase in the number of autistic children receiving teletherapy across disciplines (Allison & Levac, 2022). Additionally, an examination of access to ABA services in Nebraska found that 50 of 51 examined agencies had begun utilizing telehealth and, in response to these efforts, clients utilized more therapeutic services (Freske & Malczyk, 2021). These findings suggest that the mitigation phase, which began in June 2020, was characterized by increased access to ASD related services compared to access during the crisis phase.

The current literature on the changing nature of the COVID-19 pandemic contains little information on month-to-month changes during the first year and relies on parent-reported perceptions of disruption. While these reports are important, they are limited in that they are vulnerable to participant level subjectivity. Evaluation of an objective and repeated measure of therapy utilization would allow for the examination of how access to services changed before and through the first year of the pandemic and how these changes in access may have differed for children of different backgrounds. More objective measurement would also allow for the impact of COVID-19 to be partially distinguished from other potential mechanisms of change in ABA therapy utilization through time. For example, use of objective repeated measures allows for generally agreed upon “seasons” of lower therapy utilization (e.g. school summer and winter breaks) to be controlled for in statistical analyses of change in therapy utilization through time. This control, in turn, would allow for better estimation of the specific effects of the pandemic on changes in ABA therapy utilization.

## Racial, Ethnic, and Socio-Economic Differences in Pandemic Related Service Disruption

In addition to missing descriptions of service changes through the first year of the pandemic, there is also very limited information on how service disruption may have differed between autistic children of different racial, ethnic, and socio-economic backgrounds. Medical research identified racial and socio-economic disparities in several domains during the COVID-19 pandemic. For example, people of color and people with lower incomes faced increased risk of severe illness and death due to COVID- 19, unemployment and loss of health care coverage, less access to preventative healthcare, and higher risk of worsening mental health (CDC, 2021).

Prior to the pandemic, racial and ethnic disparities in access to ASD services were well documented. In comparison to white autistic children, Black and Latinx autistic children had significantly less access to general healthcare services (i.e., primary care, specialty medicine, social services), and Latinx children had more unmet needs with regard to psychotherapy, intensive ASD services, OT, and speech language therapy (Irvin et al., 2012; Liptak et al., 2008; Magaña et al., 2013). In schools, Latinx autistic students were significantly less likely to receive an individualized education program compared to autistic white students (Harstad et al., 2013). A systematic review of qualitative studies of racial and ethnic disparities in access to ASD services found that familial factors (e.g. financial and informational resources), cultural factors (e.g. definition of ASD, stigma), and structural factors (e.g. healthcare system policy, professional competence) each contribute to these disparities (Singh & Bunyak, 2019). The consistency of these reported disparities has led to calls to further investigate how structural racism (i.e. institutional policies that systematically favor those of certain racial/ethnic backgrounds) contributes to inequitable outcomes among autistic children of different racial and ethnic backgrounds (Broder-Fingert et al., 2020). This is especially important in light of policy changes that occurred in the context of COVID-19.

Socio-economic status had also been shown to predict different levels of ASD service access and utilization among autistic children (Liptak et al., 2008; Smith et al., 2020). Higher intensity (i.e., greater hours) of ASD-related services and utilization of more types of services was associated with higher level of parental education, higher parental job prestige, higher annual household income, and family ownership of home (Harstad et al., 2013; Patten et al., 2013; Siller et al., 2014). Additionally, studies that examined ASD service utilization among Medicaid enrollees found evidence of differential service access compared to children who obtain funding from other sources (Ruble et al., 2005; Wang et al., 2013; Yingling, 2018).

Given the comprehensive evidence of racial, ethnic, and socio-economic disparities in access to ASD services prior to the pandemic, it is crucial to examine if these disparities maintained or worsened in the context of COVID-19. Further, calls to examine structural mechanisms of disparities in access to ASD services prior to the pandemic and reports of structural obstacles to general healthcare delivery during the pandemic warrant specific exploration of the mechanisms of changing racial, ethnic, and socio-economic disparities in access to specific ASD services (such as ABA) during the COVID-19 pandemic.

## Exploration of the Mechanisms of COVID-19’s Impact on Therapy Utilization

The existing literature informs us that there was an environmental stressor (COVID-19) that led to worse outcomes for autistic children and their families (increased challenges, reduced access to services). However, there is limited exploration of the specific mechanisms through which the pandemic diminished the quality of the ASD care delivery system. Donabedian’s quality of care model has been used to examine how health delivery systems can best deliver quality care (e.g. Ayanian & Markel 2016; LoPorto, 2020). This model, often referred to as the SPO model, posits that that the quality of any healthcare system should be evaluated at the structural, process, and outcome levels. More recently, researchers have extended the model to include environmental context and patient characteristics as influential factors into the original conceptual framework (Amir et al., 2017; Qu et al., 2010).

This extended SPO model can provide a useful conceptual framework for understanding how the COVID-19 pandemic (environmental context) impacted the structure, process, and outcomes of the ASD care delivery system. While other conceptual frameworks can be used to examine disparities in health care settings (e.g. Kilbourne et al., 2006), the extended SPO model’s modular consideration of factors is preferred given the limited prior research on disparities in access to ASD services during the COVID-19 pandemic. This modular structure allows for findings from different areas (e.g. pre-pandemic disparities in ASD treatment, impacts of COVID-19 on general healthcare systems) to be combined to tell a complete story and for the consideration of how environmental stress (COVID-19) both directly and indirectly (via structure) impacted the process of therapy delivery.

In the extended SPO model, structure refers to the stable context in which care is delivered. For example, in the ASD care delivery system, structural factors include physical clinic spaces, therapy funding sources, tele-therapy equipment, therapy staff, and organization policy. Process refers to the interactions between healthcare providers and patients. In the ASD care delivery system, process includes creation of treatment plans, hours of therapy delivered, flexibility of therapy setting, responsiveness to needs of the client, and therapeutic alliance. Outcome refers to changes in the patient’s life brought on by the health system (e.g., reduction in symptoms, addition of new skills, increased quality of life; Donabedian 1980). In addition to these core domains, the extended SPO model considers how environment factors (e.g. COVID-19, federal policy) and patient characteristics (e.g. race, ethnicity, socio-economic status) influence the quality of the healthcare delivery system for individuals in specific contexts.

Research on the impacts of the COVID-19 pandemic on autistic children and their families has primarily focused on the outcomes piece of the extended SPO model (e.g. perceptions of disrupted services, challenges to daily living activities). Examination of how patient level variables (demographics) and environment level variables (phase of pandemic) effected the process level variable of hours of therapy could provide insights on the mechanisms of the pandemic’s impact on autistic children and their families. Reference to the extended SPO model provides the additional opportunity to postulate how structure level factors (institution and insurance policy) contributed to the disruption of the process of therapy delivery. Utilization of the extended SPO model in interpretation of findings allows for exploration of what parts of the ASD care delivery system failed, for whom, and inform which facets of the care delivery system to improve.

## Current Study

The current study examined how hours of ABA therapy utilization changed between March 2019 and February 2021 among autistic children in California. To assess the changing nature of the pandemic’s impacts, we examined changes in therapy utilization between three different phases of the COVID-19 pandemic: pre-pandemic, crisis, and mitigation. We hypothesized that, when child age, therapy site, and season were held constant, hours of ABA therapy utilization would significantly decrease during the crisis phase of the pandemic (March 2020 – May 2020) and then significantly increase during the mitigation phase (June 2020 – February 2021).

The current study also examined if therapy utilization differed for children of different racial or ethnic backgrounds and children with different ABA funding sources, as a proxy of socioeconomic status. The second hypothesis was that non-white participants would experience a sharper reduction in hours during the crisis phase and a lesser increase in hours during the mitigation phase compared to white, non-Latinx participants. Hypothesis three was that participants with public insurance or school district as their primary payer would experience a sharper reduction in hours during the crisis phase and a lesser increase in hours during the mitigation phase compared to participants with private insurance as their primary payer. If our findings supported the 2nd and 3rd hypotheses, we planned to run exploratory analyses examining a potential three way interaction between time, child race/ethnicity, and child primary payer.

Finally, findings were interpreted using the extended SPO model as a conceptual framework. This conceptualization allowed for findings to be interpreted within the context of the larger ASD care delivery system and allowed for potential inferences to be made about how different facets of the ASD care delivery system were impacted by the COVID-19 pandemic.

## Method

### Overview

This study utilized retrospective, de-identified data from one multisite ABA agency that serves autistic children. Data consisted of guardian-reported client demographics and detailed client therapy logs. Data came from each of the agency’s five branches (Contra Costa County, East Los Angeles, South Bay in Los Angeles County, North Los Angeles, and West Los Angeles), representing a racially and socioeconomically diverse set of geographical regions in California.

### Participants

Participants included in the sample consisted of autistic children who received ABA therapy from this agency, at any of the branches, between March 1, 2019 and February 28, 2021. Because our aim was to examine change in therapy utilization across phases of the pandemic, participants were excluded from the sample if they ended treatment prior to March 2020. These cases were identified by having zero hours of therapy as of February 2020 or sooner with no return to therapy during the remainder of the study period. Additionally, potential participants were excluded if they received zero funding for services from private insurance, public insurance, or school districts (e.g. those with exclusive funding from regional centers). These funding sources were excluded from the sample because they are typically transient (i.e. provide funding for six months or less) and the purpose of the study was to examine changes in long term ABA utilization.

Of the 313 participant records that were obtained, 13 were excluded for ending treatment prior to March 2020 and 17 were excluded for having exclusive funding from regional centers (and therefore, not private insurance, public insurance, or school district). After exclusion, total sample size consisted of 283 children whose ages ranged from one to fourteen years old (Mean = 5.68, SD = 2.99). Table 1 summarizes the demographic characteristics of the sample including participant race/ethnicity, gender, and therapy site.

**Table 1:** *Characteristics of Study Participants*

	N	Percent
**Race/Ethnicity**		
Asian, non-Latinx	61	21.6%
Black, non-Latinx	19	6.7%
Latinx and any Race	52	18.4%
White, non-Latinx	82	29%
Other of Mixed Race/Ethnicity	6	2.1%
Not Reported	62	22.3%
**Gender**		
Male	209	73.9%
Female	63	22.3%
Not Reported	11	3.9%
**Therapy Site**		
Contra Costa County	57	20.1%
East Los Angeles	41	14.5%
North Los Angeles	56	19.8%
South Bay, Los Angeles County	76	26.9%
West Los Angeles	53	18.7%

## Measures

### Hours of ABA Therapy

Total hours of direct-service (excluding supervision, preparation, etc.) ABA therapy were calculated for each participant for each month within the study time period. Hours of all settings, including telehealth delivered hours, were including in calculations.

The specific variable, monthly hours of ABA, was chosen for this study for several reasons. First, ABA was chosen over other evidence-based therapy modalities because of its relative accessibility and common utilization across development (Hyman et al., 2020). Autistic children are recommended 20–40 h of ABA therapy a week prior to kindergarten at which point treatment setting and goals shift to match developmental and academic needs (Board, 2014). The accessibility of ABA in the US is supported by funding through public institutions such as school districts, regional centers, private health insurance, and Medicaid. While other therapy modalities for ASD were likely impacted, ABA was chosen because of its common utilization.

Additionally, a quantitative outcome variable (hours per month) was chosen over a more descriptive variable (e.g. client satisfaction, goal attainment) in order to make large scale comparisons on a single, generalizable variable. Specific outcomes of therapy vary greatly between autistic children and, because of this variability, there is little consensus on how to measure outcomes across children in a standardized way (Matson & Rieske, 2014). Hours of therapy are objectively measurable and allow for unbiased comparisons across autistic individuals. Despite the lack of descriptive outcomes, previous research suggests that, on average, more hours of ABA therapy leads to more treatment gains, including improved cognitive abilities, adaptive functioning, language skills, social skills, and quality of life (Linstead et al., 2017; Magiata, 2012). While some nuance may be lost, the examination of hours of therapy allows for large group comparisons that have yet to be explored in the literature.

### Race/Ethnicity

The data received from the agency included the child’s parent-reported racial/ethnic identity. Based on this information, participants were categorized as Asian, Non-Latinx (n = 61, 21.6%); white, Non-Latinx (n = 82, 29%); or other racial/ethnic identity (n = 77, 27.2%). Participants were grouped in the “racial/ethnic identity other than Asian or white” group if they did not meet group criteria for the Asian, non-Latinx group or the white, non-Latinx group. For example, this group included those who identified as Black, African American, Latinx, Hispanic, Chicano, Native American, “other”, and “mixed”.

The singular “other group” was chosen instead of three distinct groups (i.e. Latinx, Black, and Other) in order to maximize analytic power. However, in order to ensure that differences were not lost within the “other” group, we ran analyses with five groups (Asian, Black, Latinx, white, Other) and compared results with analyses run with three groups. No differences between analyses were found so we proceeded with three levels of race/ethnicity.

### Primary Payer

Participants were grouped into one of three categories of primary payer: private insurance as primary payer, public insurance as primary payer, or school district as primary payer. Because any one child could receive funding from multiple sources (e.g., both private insurance and school), the following system was developed to categorize each participant into one of the three primary payer categories. Participants were grouped into the private insurance group if more than 50% of their hours were funded by a private insurer (n = 174); into the public insurance group if more than 50% of their hours were funded by a public insurer (n = 39); and into the school district group if more than 50% of their hours were funded by a school district (n = 70).

### Season

Given that young children’s schedules in the US are determined by the school year, we added a “season” covariate in order to control for school-related fluctuations in therapy utilization. We coded each month as an “on month”, or months when therapy is at regular intensity (i.e. school year months) or an “off month”, months when a child’s therapy is reduced (i.e. summer break, winter break). January through May, September, and October were coded as “on months” and June through August, November, and December were coded as “off months”. The accuracy of this pattern was confirmed via visual inspection of a spaghetti plot.

## Procedure

All study procedures were reviewed and approved by the University of Southern California Institutional Review Board. First, the principal investigator met with the community-based ABA agency in order to develop a mutually effective study protocol. The agency then selected a team of internal agency employees to gather clinical data from their internal system, de-identify all clinical records (aside from date of birth), and transfer data to the research team. Data was included for demographics (date of birth, reported race/ethnicity, and reported sex/gender) and ABA therapy delivery information (therapy session duration, therapy session funder, therapy session setting) for the time period between March 1, 2019 through February 28th, 2021.

### Data Analysis

The first step of analysis was to examine descriptive statistics and missing data. Then, we prepared to run a multilevel model by conducting an unconditional model and calculating the interclass correlation and design effects. The final step of preparation was to visually examine phase changes using a spaghetti plot and code three distinct phases in time in order to run a piecewise growth model. Hypotheses were then evaluated using four separate piecewise growth multilevel models. The first model evaluated how time predicted change in therapy utilization at each of the three phases when covariates were held constant. The following three models utilized the same primary predictor, covariates, and phases and only differed in inclusion of moderators. The second model evaluated the moderating effects of child race and ethnicity, the third evaluated the moderating effects of child primary payer, and the fourth evaluated the effects of a three way interaction between time, child race/ethnicity, and child primary payer. All analyses were conducted using R Studio (2020).

## Results

Prior to hypothesis testing, mean and standard deviations were calculated for each subgroup of participants for the entire time period and for each phase (Table 2). 180 participants had data for all 24 timepoints. The remaining 103 participants began ABA therapy after the March 2019 start point and, therefore, did not have data for all 24 timepoints. Once a participant began treatment, there were no missing values for any of the following months. All participants were included in the models, regardless of therapy start date, because the proportion of missing data was similar for each subgroup and multi-level modeling readily handles models with missing data or uneven data structure.

**Table 2:** *Descriptive Statistics of Hours of Therapy Utilization*

Group	All Time Mean(SD)	Phase 1 Mean(SD)	Phase 2 Mean(SD)	Phase 3 Mean(SD)
All	59.92 (41.77)	76.93 (43.13)	35.94 (33.06)	48.81 (34.82)
White, non-Latinx	65.82 (42.37)	83.01 (41.78)	37.41 (34.63)	54.21 (36.24)
Asian, non-Latinx	54.60 (38.92)	68.16 (42.45)	38.10 (32.84)	44.48 (30.70)
Other race/ethnicity	58.93 (42.26)	78.04 (44.00)	33.96 (30.35)	45.33 (33.24)
Private Insurance	57.22 (39.29)	68.97 (40.08)	37.55 (33.40)	50.81 (36.22)
Public Insurance	48.09 (36.40)	62.13 (41.04)	31.31 (29.73)	39.01 (27.39)
School District	71.20 (46.46)	98.74 (41.73)	34.74 (33.73)	49.43 (34.26)

*Note. The “Other race/ethnicity” group consists of participants who did not identify white or Asian and all participants, regardless of race, who identified as Latinx*

### Preliminary Analysis

The data provided by the ABA agency was fitted to a two-tier hierarchical multi-level model with repeated measures of monthly therapy utilization (level 1; n = 5,490) nested within individual children (level 2; n = 283). Level 1 variables were time variant and included monthly hours of ABA therapy and season. Level 2 variables consisted of time invariant information about the individual child: race/ethnicity, primary payer, gender, age, and therapy site.

Before testing hypotheses, we ran an unconditional model to examine how hours of therapy utilization were predicted by variance between participants and to calculate interclass correlation. The ICC was 0.374, indicating that a 37.4% of the variance in therapy utilization was due to differences between participants as opposed to differences between timepoints. The design effect was 9.625. Because the design effect was greater than 2, we decided to proceed with multilevel modeling in order to account for the clustering structure of the data (Lai & Kwok, 2015).

### Phase Identification for Piecewise Growth Model

Given that we hypothesized a curvilinear relationship between time and hours of service (i.e. initial reduction followed by increase), we utilized a piecewise growth model in order to evaluate different slopes for different phases. Piecewise growth modeling is a modeling method that breaks up a repeated measure span (e.g. time 0 through time 10) into distinct phases (e.g. time 0–5, time 6–10). In piecewise growth modeling, at least one turning point is selected in order to create phases. Piecewise growth modeling allows for the modeling of distinct linear slopes for each phase, rather than one linear slope across the entire repeated measure time period.

In order to confirm the theoretical phase periods described previously (pre-pandemic, crisis, and mitigation), we ran a spaghetti plot in order to visualize average change in hours of therapy for the current study’s sample. The spaghetti plot was also used to confirm the accuracy of the months chosen as “off months” in the season covariate by examining changes in the 11 months prior to the onset of the pandemic. Visual inspection revelated that, between March 2019 and February 2020, there were marked reductions in therapy utilization for the months of June, July, August, November, and December, thus confirming the months chosen to be coded as “off” in the season covariate. Once the season changes were accounted for, visual inspection of the plot confirmed the theoretical phases of change we hypothesized. The pre-pandemic time period was relatively stable and lasted between March 2019 and February 2020. Between March 2020 and May 2020, it appeared that hours of therapy utilization decreased and broke away from the pattern seen between March and May 2019. In June 2020, hours of therapy appeared to increase and match the pattern of month-to-month change seen between June 2019 and February 2020. Based on this information, we proceeded with the piecewise growth model in order to attain three separate linear slopes for three phases of time: March 2019 – February 2020 (pre-pandemic), March 2020 – May 2020 (crisis), and June 2020 – February 2021 (mitigation).

In the piecewise growth model, the first slope was estimated by coding months in the following scheme: March 2019 = 0, April 2019 = 1, May 2019 = 2, …, January 2020 = 10, February 2020 through February 2021 = 11. The first slope estimated change in hours of ABA therapy between March 2019 and February 2020, or 11 months prior to the onset of the COVID-19 pandemic. The second slope was estimated with the following scheme: March 2019 – February 2020 = 0, March 2020 = 1, April 2020 = 2, May 2020 through February 2021 = 3. The second slope estimated change in hours of ABA therapy between March 2020 and May 2020, or the crisis phase of the pandemic. The third slope was estimated with the following scheme: March 2019 – May 2020 = 0, June 2020 = 1, July 2020 = 2, …, January 2021 = 8, February 2021 = 9. The third slope estimated change in hours of ABA therapy between June 2020 and February 2021, or the mitigation phase of the pandemic.

### Hypothesis Testing

We evaluated hypotheses using four piecewise growth multi-level models. The first model evaluated how time predicted change in therapy utilization at each phase when season and participant age, gender, and therapy site were held constant. Random slopes for each phase were used for each participant in order to account for between participant differences in change in therapy utilization through time. We performed the multilevel model with the Generalized Linear Mixed Models using Template Model Builder (glmTMMB) package which fits models using maximum likelihood estimation (Brooks et al., 2017).

Results of the first model are summarized in Table 3. Time significantly predicted change in hours of therapy utilization at each phase. Time in phase 1 (pre-pandemic) was associated with a decrease of 1.54 h per month (SE = 0.26, p < .001), time in phase 2 (crisis) was associated with a decrease of 10.65 h per month (SE = 1.05, p < .001), and time in phase 3 (mitigation) was associated with an increase of 2.39 h per month (SE = 0.28, p < .001). The intercept, 90.61 (SE = 4.79, p < .001), indicates that participants received an average of 90.61 h of therapy in March 2019. The three covariates, season, age, and therapy site, were all significant predictors of change in hours of therapy utilization through time.

**Table 3:** *Results of Main Effects Model (Model 1)*

	Estimate	SE	p
Intercept	90.61	4.79	< 0.001***
Phase 1	-1.54	0.26	< 0.001***
Phase 2	-10.65	1.05	< 0.001***
Phase 3	2.39	0.28	< 0.001***
Season	-5.75	0.60	< 0.001***
Age	-1.31	0.53	0.013 *
Site 1	2.01	5.09	0.693
Site 2	1.83	4.49	0.684
Site 3	-4.88	4.61	0.291
Site 4	19.28	4.99	< 0.001***

*Note. Intercept = mean number of hours of therapy when all predictor variables in the model are equal to 0. Phase 1 = pre-pandemic phase in time, March 2019 – February 2020. Phase 2 = crisis phase in time, March 2020 – May 2020. Phase 3 = mitigation phase in time, June 2020- February 2021. Season = on month of therapy coded as 1, off month of therapy coded as 0. Site refers to agency branch: Site 0 = Contra Costa, Site 1 = East LA; Site 2 = Southwest LA, Site 3 = North LA, Site 4 = West LA.*

*p < .05. **p < .01. ***p < .001

**Fig. 1:**
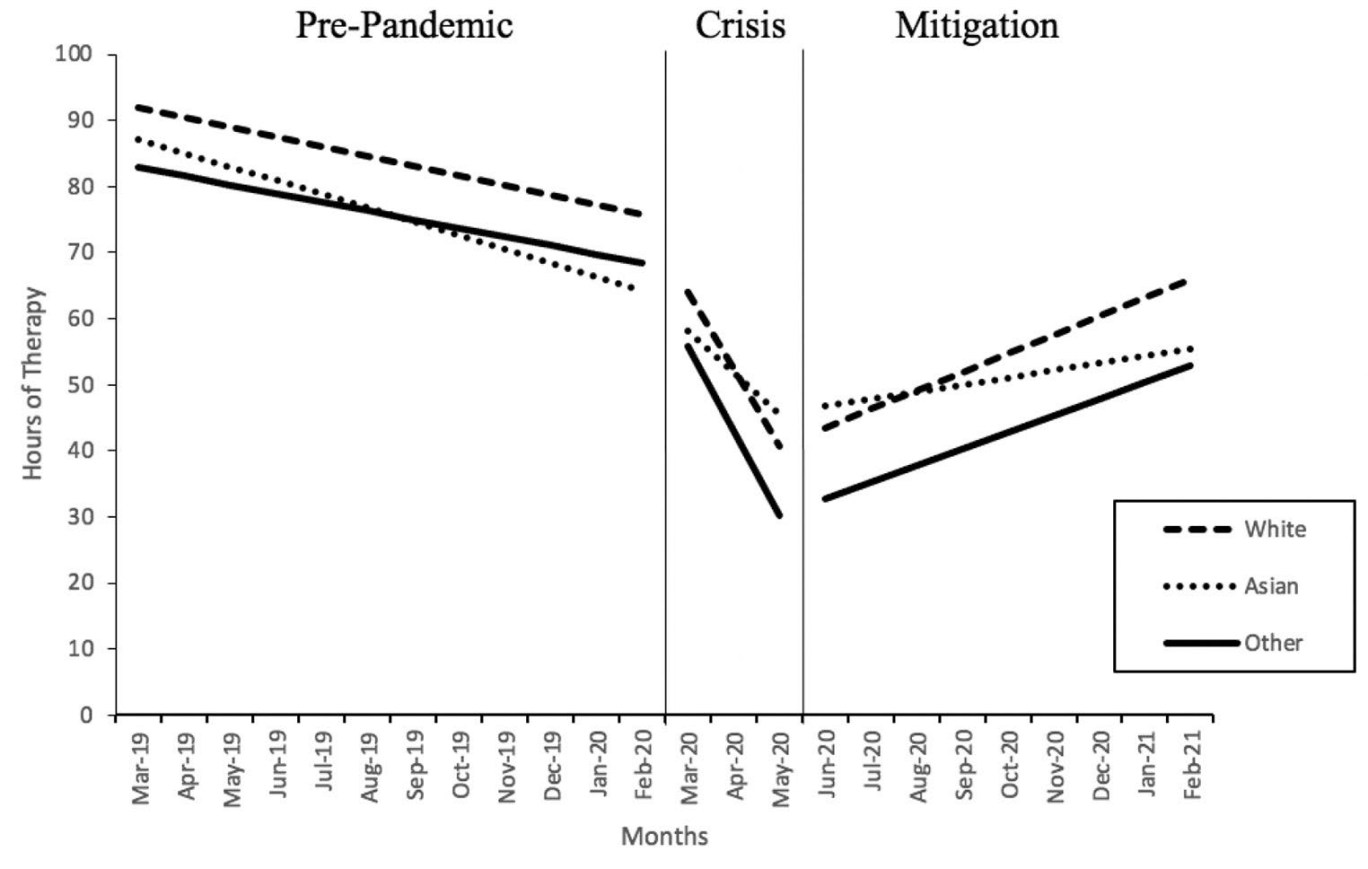
*Results of the piecewise growth multilevel model of time predicting hours of therapy utilization at three phases with child race/ethnicity as a moderating variable*

The second, third, and fourth models shared identical structure to the first model aside from their inclusion of additional moderators. Results of the second, third, and fourth models are summarized in Table 4. The second model evaluated the moderating effects of participant race and ethnicity on how time predicted change in therapy utilization at each phase (Fig. 1). There was no significant difference between white participants and other race/ethnicity participants in how time predicted change in hours of therapy utilization at any of the three phases. There was no significant difference in trajectory of therapy utilization between Asian and white participants during phase 1 (pre-pandemic) and 2 (crisis). During phase 3, Asian racial/ethnic identity significantly predicted a smaller increase in hours of therapy utilization through time compared to white racial/ethnic identity (estimate = -1.69, SE = 0.41, p < .05). In other words, for each month in phase 3, white participant’s hours of therapy increased by 1.69 more hours/month compared to Asian participants.

**Table 4:** *Results of Moderator Effect Model (Models 2, 3, and 4)*

	Phase 1 (Pre-Pandemic)			Phase 2 (Crisis)			Phase 3 (Mitigation)			
	Estimate	SE	p	Estimate	SE	p	Estimate		SE	p
**Model 1: Main Effects of Time (with covariates)**										
Intercept	90.61	4.79	< 0.001***							
Time	-1.54	0.26	< 0.001***	-10.65	1.05	< 0.001***	2.39	0.28		< 0.001***
**Model 2: Interaction between Race/Ethnicity and Time**										
Intercept	90.98	6.64	< 0.001***							
White	-1.52	0.46	< 0.01**	-11.57	1.79	< 0.001***	2.76	0.48		< 0.001***
Asian	-0.51	0.73	0.481	4.98	2.77	0.072	-1.69	0.74		0.023*
Other	0.24	0.68	0.721	-1.36	2.61	0.604	-0.20	0.70		0.771
**Model 3: Interaction between Primary Payer and Time**										
Intercept	90.24	5.15	< 0.001***							
Private	− 2.06	0.34	< 0.001***	-5.69	1.16	< 0.001***	1.60	0.35		< 0.001***
Public	− 0.11	0.79	0.887	1.27	2.67	0.631	-0.81	0.79		0.307
School	1.88	0.57	< 0.001***	-18.43	2.02	< 0.001***	3.31	0.62		< 0.001***
**Model 4: Interaction between Race, Primary Payer, and Time**										
Intercept	88.66	7.63	< 0.001***							
White/Private	-1.87	0.63	< 0.01**	-5.80	2.06	< 0.01**	1.92	0.61		< 0.01**
White/Public	-0.92	1.40	0.513	-0.15	4.70	0.974	-0.96	1.42		0.498
White/School	1.61	1.02	0.116	-19.41	3.50	< 0.001***	3.37	1.06		< 0.01**
Asian/Private	-056	0.89	0.529	2.43	2.96	0410	-0.63	0.88		0.474
Asian/Public	0.45	2.36	0.848	1.18	7.68	0.877	-1.40	2.25		0.532
Asian/School	1.21	1.87	0.518	-2.28	6.23	0.714	-3.11	1.90		0.102
Other/Private	-0.01	0.94	0.99	-3.80	3.10	0.220	0.06	0.92		0.952
Other/Public	1.21	1.98	0.541	3.38	6.49	0.603	0.58	1.93		0.765
Other/School	0.19	1.51	0.899	6.47	5.13	0.207	-1.14	1.56		0.466

*Note. Intercept = mean number of hours of therapy when all predictor variables in the model are equal to 0. Phase 1 = pre-pandemic phase in time, March 2019 – February 2020. Phase 2 = crisis phase in time, March 2020 – May 2020. Phase 3 = mitigation phase in time, June 2020- February 2021. White = white, non-Latinx; Asian = Asian, non-Latinx; Other = all racial identities aside from white and Asian and all who identified as Latinx. Private = private insurance as primary payer of therapy; Public = public insurance as primary payer of therapy; School = School district as primary payer of therapy*

*Repeating the analysis with five groups for race/ethnicity (white, Asian, Black, Latinx, Other) and five groups for primary payer (private insurance, public insurance, school district, private insurance and school district, and public insurance and school district) led to similar results*

*p < .05. **p < .01. ***p < .001

**Fig. 2:**
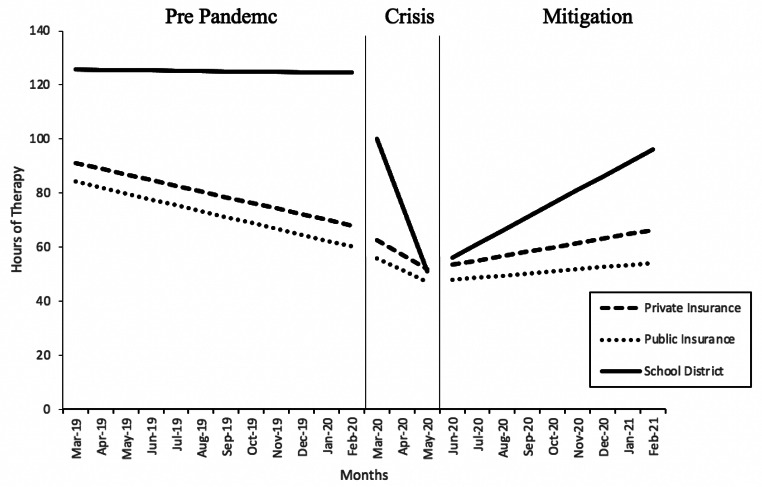
*Results of the piecewise growth multilevel model of time predicting hours of therapy utilization at three phases with child primary payer as a moderating variable*

In the third model, we removed participant race/ethnicity and included participant primary payer in order to evaluate the moderating effects of participant primary payer on how time predicted change in therapy utilization at each phase (Fig. 2). There was no significant difference in change of hours of therapy utilization through time between private insurance and public insurance funded children at any of the three phases. School district funded children had a significantly different trajectory of hours through time at each phase compared to private insurance funded children. During phase 1 (pre-pandemic), school district as primary payer significantly predicted a lesser reduction of therapy utilization through time compared to private insurance as primary payer (estimate = 1.88, SE = 0.5, 7, p < .001). During phase 2 (crisis), school district funding significantly predicted a sharper decrease in hours of therapy utilization through time by an average of -18.43 h/month (SE = 2.02, p < .001) compared to private insurance funding. During phase 3 (mitigation), school district funding significantly predicted a sharper increase in hours of therapy utilization through time by an average of 3.31 h/month (SE = 0.62, p < .001) compared to private insurance funding.

The fourth and final model included both participant race/ethnicity and primary payer in order to evaluate if there was a three way interaction between time, participant race/ethnicity, and participant primary payer that predicted change in therapy utilization at each phase. We found that there was no significant three way interaction at any of the three phases.

## Discussion

The purpose of the current study was to examine (a) how autistic children’s ABA therapy utilization changed through the COVID-19 pandemic and, (b) examine if that relationship was moderated by child’s racial/ethnic identity and/or primary funding source. We found that hours of therapy utilization dramatically fell during the crisis phase of the pandemic and, while hours increased during the mitigation phase, the increase did not make up for the loss of hours in the crisis phase. Moderator analyses revealed that children with school district funding, who may represent the most vulnerable, experienced differential impact during all three phases. Finally, Asian participants had diminished recovery of hours compared to participants of all other races and ethnicities. Further discussion of these findings through the lens of the extended SPO model will be considered in order to contextualize findings to the larger ASD care delivery system.

## Change in Hours of ABA Therapy Over Time

We first examined how hours of ASD service utilization changed through time within three phases of the COVID-19 pandemic: pre-pandemic, crisis, and mitigation. We found that, prior to the onset of the pandemic, hours of therapy utilization decreased by 1.5 h per month. This small yet statistically significant change likely reflects a combination of seasonal effects that were not captured by the season covariate and individual-level reductions in therapy utilization due to goal progression. This reduction is not unexpected given that ABA therapy utilization, generally, starts with high intensity and gradually reduces as treatment goals are met (Board, 2014). While the reductions in the pre-pandemic and crisis phases were both statistically significant, the reduction in the crisis phase was approximately seven times steeper than the pre-pandemic phase. This indicates that a change in therapy utilization during the crisis phase was due to more than normative fluctuation.

As hypothesized, we found a statistically significant reduction in ASD service hours during the crisis phase and a significant increase in service hours during the mitigation phase. While changes in therapy utilization were significant and in opposite directions between the crisis and mitigation phases, the magnitude of change was much larger during the crisis phase. Participants lost an average of 10.65 h per month in the first three months of the pandemic and gained an average of 2.38 h per month in the following 9 months. This means that it took about 5 months in the mitigation phase to recover the hours of therapy lost in about 1 month of the crisis phase. Together, these findings suggest that autistic children’s utilization of ABA therapy was reduced at the start of the pandemic and had not fully recovered to pre-pandemic levels a full year later.

Contextualized to the extended SPO model, our findings suggest that an environmental factor (COVID-19) impeded the process of ABA therapy delivery both indirectly (via structural limitations) and directly. Given the extended SPO’s model conceptualization that high quality structure is a prerequisite for high quality process (e.g. therapy hours delivered), structural disruptions (e.g. reductions in personnel, loss of therapy space, and loss of funding) likely contributed to the reductions in therapy utilization found in our study (Donabedian, 1980).

While we were unable to find examinations of ABA workforce changes during the pandemic, reductions in therapist workforce have been recorded in several other disciplines of psychology (Abrams, 2020; Oster et al., 2020). It is possible that the field of ABA experienced a similar loss of personnel during the pandemic which would have led to less availability for agencies to schedule clients. Additionally, given that many families chose to reduce in-person ABA to minimize risk of exposure to COVID-19, continuation of therapy heavily relied on access to tele-therapy technology (Rodriguez, 2020). It is possible that structural limitations to setting up tele-health visits (e.g. limitations in technology access, financial resources, and payer/provider policies) further impeded the agency’s ability to deliver ABA therapy. Finally, financial strain across funding sources may have led to a reduction in the number of hours autistic children were approved to receive by funding sources.

In addition to these structural limitations, it is possible that changes to process-level ABA delivery protocols further contributed to the reductions in therapy utilization found in our study. For example, therapists may not have been able to deliver an ideal care plan because of inconsistent therapy schedules, masking, distancing during in-person sessions, and limited treatment modalities in a tele-therapy setting. This lower quality process is evidenced by high levels of dissatisfaction with ASD related services and mixed reports of the acceptability of tele-therapy among autistic children during the COVID-19 pandemic (Bhat, 2021; Freske & Malczyk, 2021; Murphy et al., 2021). It is possible that this dissatisfaction with therapy led clinicians and families to decide to reduce therapy utilization during the COVID-19 pandemic.

Regardless of the precise cause of disruption to the process of ABA therapy delivery, the extended SPO model conceptualizes that high quality outcomes rely on high quality process. Therefore, the finding that hours of therapy were significantly disrupted for a full year following the onset of the pandemic may suggest that short and long term outcomes of autistic children were also impacted. Previous literature on the effects of delaying the start of early intensive ABA intervention found that autistic children who began treatment earlier in life experienced more positive outcomes including higher IQ gain and higher likelihood of placement in a general education classroom compared to children who started treatment later in life (Tarbox et al., 2013). This suggests that autistic children who experienced long term disruption to ABA therapy may experience worse outcomes due to the disruption. This theoretical concern is consistent with preliminary findings that autistic children have experienced a decline in functioning across several domains since the onset of the COVID-19 pandemic (Allison & Levac, 2022). In combination with our findings, this literature suggests that the disruption to ASD related services during the first year of the pandemic may have negatively impacted treatment outcomes for autistic children.

## Differential Impact of COVID-19 on Hours of ABA Therapy by Child’s Race and Ethnicity

Examination of the moderating effects of child race/ethnicity on how hours of therapy utilization changed during the pandemic showed no differences in the rate of therapy utilization between children of different races or ethnicities during the pre-pandemic or crisis phases. These findings suggest that the reduction in therapy utilization brought on by the COVID-19 pandemic was not better or worse for children of different races or ethnicities.

We did, however, find racial differences in the rate of therapy recovery during the mitigation phase. Compared to white children and children of other races or ethnicities, Asian children had a relatively smaller monthly increase in hours of therapy utilization (+ 1.08 h/month for Asian compared to + 2.76 h/month for white). One possible mechanism is that clinicians and Asian American and Pacific Islander (AAPI) families had cultural and linguistic differences (e.g. misunderstandings about the meaning of the child’s disability, treatment goals, and role of caregivers in treatment) that created barriers to accessing services, as has been demonstrated in pre-pandemic literature (Harry, 2008; Lo, 2008). Additionally, AAPI parents of children with disabilities have been shown to have difficulty advocating for their children in individualized education program (IEP) meetings due to language barriers and misunderstandings of their role in service planning (Bacon & Causton-Theoharis, 2013; Lo, 2008). Given that amelioration of therapy hours likely required adaptations to treatment plans and coordination with parents (e.g. parent facilitated tele-therapy), it is possible that cultural and linguistic differences between AAPI families and providers led AAPI autistic children to have a slower increase in therapy utilization during the mitigation phase of the pandemic.

Additionally, the environmental context of rising discrimination and hate incidents towards AAPI people in the US may have contributed to the disparity observed in our findings. In the US, societal perception that COVID-19 was the fault of China led to rises in negative sentiments and hate incidents towards all AAPI people (Jeung et al., 2021; Ng, 2021; Pew, 2020). It is possible that negative sentiments towards AAPI families of autistic children led ASD service providing institutions and clinicians to reduce the offered hours of therapy. For example, the biased belief that interactions with AAPI people would increase risk of exposure to COVID-19 may have led clinicians to avoid in-home sessions with AAPI autistic children. Additionally, higher levels of stress and anxiety experienced by AAPI people in the US due to the rise in hate incidents may have led to AAPI parents of autistic children to be more reserved with self-advocacy in order to avoid potential discriminatory incidents (Lee & Waters, 2021). While it is difficult to draw a direct line between the rise of negative sentiments towards AAPI people in the US and the results of our study, the prominence of the rise of anti-AAPI sentiments makes it unlikely that this environmental context had no impact on our findings.

Our finding that Asian children had a relatively slower recovery of hours compared to children of other races and ethnicities may indicate that AAPI autistic children may have been left behind to some degree in mitigation efforts. Special attention should be paid to assure that therapy utilization among AAPI autistic children has since been recovered. Additionally, it should be noted that, similar to AAPI people, Black and Latinx people in the US faced cultural and linguistic barriers to accessing ASD services prior to the pandemic and that Black people in the US faced a rise in hate incidents in 2020 (Federal Bureau of Investigation, 2021; Singh & Bunyak 2019). It is possible that these factors also led to worse disruption to ABA therapy utilization for these populations, but the limited representation of Black and Latinx participants in our sample may have masked a true effect. Therefore, special attention should be given to Black and Latinx autistic children as well in order to monitor access to ABA services.

## Differential Impact of COVID-19 on Hours of ABA Therapy by Child’s Primary Payer

Our moderator analyses revealed that autistic children who received primary funding from school districts had different rates of change in therapy utilization through time at each of the three phases. In the pre-pandemic phase, school district funded children had relatively less variability in hours of therapy utilization, month to month, compared to children with other funding sources. This difference may reflect that children with school district funding had relatively more consistency in treatment prior to the pandemic and/or the season covariate captured seasonal variation among this payer group better than it did for other groups.

The deviation of therapy utilization among school district funded children compared to children with other funding sources became more stark in the two pandemic phases. In the crisis phase, school district funded children had a sharper reduction in therapy utilization through time (-24.11 h/month) relative to private insurance funded participants (-5.68 h/month) and public insurance funded participants (-4.41 h/month). The differential trajectory maintained in the mitigation phase with school district funded children experiencing a sharper monthly increase in hours of therapy (4.91 h/month) relative to private insurance funded (1.60 h/month) and public insurance funded (0.80 h/month) children. While school district funded children did experience a sharper increase during the mitigation phase, the relative magnitude of change between the crisis and mitigation phases indicates that school district funded children, especially, lost a substantial amount of support during the first year of the pandemic relative to children with funding from other sources.

Examination of the structure and process variables specific to school district funding may provide insights as to why school district funded children had their hours more severely impacted during the pandemic. While schools are mandated to distribute resources to ensure that students with disabilities receive a free and appropriate education (FAPE), studies from prior to the pandemic indicate that school districts may not provide the mandated level and quality of services (Gelbar et al., 2018; Ruble et al., 2010). It is possible that systematic obstacles to providing FAPE for students with disabilities continued or worsened during the pandemic and led to the decrease in therapy utilization experienced by autistic children with school district funding. In fact, a recent federal investigation into the Los Angeles Unified School District (LAUSD), where many of our participants received funding, concluded that LAUSD failed to provide FAPE to students with disabilities during the COVID-19 pandemic (US Department of Education, 2022). The investigation, conducted by the US Department of Education Office for Civil Rights, found that LAUSD limited services provided to students with disabilities for reasons other than the students’ educational needs, reduced student services without appropriate prior evaluation or consultation, failed to accurately track services provided to children with disabilities, and failed to implement a plan to remediate instances of unjustly lost services. This suggests that the relatively worse recovery of therapy utilization among school district funded autistic children may be due to school districts’ structural limitations (i.e. funding, personnel, prioritization of other issues).

Limited resources at the structural level may have also affected the quality of the therapy delivery process for autistic children experiencing remote learning. For example, the structure of a virtual classroom may have made it more difficult for therapists to support their clients without disclosing the client’s identity and violating the US Health and Portability Accountability Act (HIPAA, 1996). Lower quality of school-based ASD services is evidenced by the finding that parent-reported satisfaction with their child’s therapeutic services during the pandemic was negatively predicted by utilization of school-based services (Murphy et al., 2021). As described previously, lower quality of therapy may then have led clinicians and families to decide to reduce the intensity of therapy for some autistic children.

The finding that school district funded participants experienced greater reduction in service hours relative to participants who received funding from other sources may indicate that those with less resources were especially impacted. Because school district funding for support for students with disabilities is federally mandated by IDEA, it is possible that school district funding is the only source of support for autistic children with limited resources (e.g. those with limited or no access to medical insurance; IDEA, 2004). Therefore, our findings may suggest that children with the least resources, who depend on school district support, had the most severe disruption to services during the initial months of the pandemic relative to children with more comprehensive access to supports.

Finally, the finding that there was no significant difference in impact of the pandemic between private insurance funded and public insurance funded children is also of note. The lack of difference between private insurance and public insurance funding, in this sample, may suggest similar access to ABA services between these two funding pathways during times of stress. This finding is encouraging, given that publicly funded insurance is likely a proxy for families from lower socioeconomic backgrounds, relative to private insurance. However, while there were no significant differences between these groups, it should be noted that there was a trend of less utilization at all phases among public insurance funded children compared to private insurance funded children (see Table 2). This trend suggests that further investigation (e.g. larger sample, larger geographic space) into differences between these groups in ABA therapy utilization may be warranted.

## Three Way Interaction

Our exploratory analysis of three way interactions between time, child race/ethnicity, and child primary payer yielded no significant results. This finding indicates that children of different races and ethnicities were perhaps not treated differently by their funding sources with regard to utilization of ABA therapy during the first year of the pandemic. This finding contributes to the pre-existing, yet limited, mixed discourse on the interaction between health policy and racial and ethnic disparities in access to ASD services. Our findings align with a pre-pandemic study that found changes in health insurance coverage policies brought on by state mandates did not improve nor worsen racial and ethnic disparities in access to ASD services (Doshi et al., 2017). However, another larger study found that, among those with consistent sources of care (an indicator of high quality healthcare coverage), white children received earlier ASD diagnosis compared to African American children (Emerson et al., 2016). It is possible that Emerson et. al.’s study’s much larger sample (n = > 96,000) and control of more confounding variables allowed for true interactions to be demonstrated. While our findings did not indicate an interaction between racial/ethnic identity and primary payer, it is possible that replications with larger samples may produce different findings.

## Limitations

One limitation of the current study is that the sample is restricted to autistic children who receive ABA therapy services from one multi-site agency in California. The five clinics whose data were analyzed for the study serve a socially and geographically diverse collection of California, the most populous state in the US. Still, it is entirely possible that unknown variables systematically biased the families who were able to access services through the one agency and therefore participate in the current study sample.

Second, we did not explicitly examine quality of ABA therapy outcomes. Implications regarding the quality of ABA therapy outcomes made in this study rely on the evidence-based assumption that more hours of ABA therapy are associated with better outcomes among autistic children. While this association is heavily supported in the literature (Linstead et al., 2017; Magiata, 2012), the connection between hours of ABA therapy and positive outcomes could have been fortified if the study included parent, child, therapist, and teacher perceptions of ABA therapy quality throughout the COVID-19 pandemic, and/or other measures of learning (e.g., standardized assessments of skill development, ASD symptoms, etc.).

Finally, the use of retrospective clinical data limited our ability to systematically collect participant demographic data. We did not have race or ethnicity data for 22% of our participants and it is possible that the addition of this data would have affected the results of the analyses. Additionally, the limited representation in the sample and limited racial/ethnic information in the data led to grouping of all participants who identified as Asian regardless of ethnicity into one category and the grouping of Black, Latinx, and Other racial identity into one category. This broad categorization may have led to masking of differential impacts of people of different backgrounds within each category.

## Future Directions

Future research could extend on the results of the current study by expanding the sample size, geographic region, ASD service type, and study timeline. For example, how did therapy utilization change in other states or in other countries? Were other ASD services such as occupational therapy, speech language therapy, and counseling effected similarly to ABA therapy? How did therapy utilization change with the prominence of the COVID-19 vaccine in early 2021? The current study provides an important foundation to understanding the pandemic’s impacts on ASD service delivery, but further research could potentially provide a clearer picture of the pandemic’s impact on ASD service utilization.

Additionally, the current study covers one facet of ASD care delivery system, ABA therapy utilization. There are several other areas of ASD care such as access to diagnostic evaluations and enrollment into treatment that our findings do not speak to. For example, we found no differences between white participants and other (i.e., Black, Latinx) participants in rates of therapy utilization, but we do not know if there were differences between these groups in the quality of services received. Treatment utilization is an important aspect of the ASD care system, but several other areas of the ASD care system may have been affected.

Finally, the current study’s use of the extended SPO model allowed for the theoretical exploration of the potential long term impacts of service disruption on autistic children. However, theoretical impacts based on literature prior to the pandemic are not sufficient in understanding how COVID-19’s disruption of ASD services actually impacted autistic children. Future researchers should consider examining functional and emotional outcomes among autistic children whose services were disrupted to different degrees during the COVID-19 pandemic.

## Conclusion

This study expands upon previous literature by examining the ways in which the COVID-19 pandemic and associated restrictions impacted therapeutic services among autistic children. These findings carry crucial implications for autistic children who live through unexpected world events and significant disruptions to school and health care systems. These findings should be used to inform the extent to which children whose therapy was disrupted during the COVID-19 pandemic should receive additional support to make up for therapy time lost. Additionally, findings should be used to inform potential areas of improvement to the ASD care system so that future world events result in less negative impact on autistic children. For example, at the structural level, the now existing COVID-19 safety policies related to funding, tele-health utilization, and in-person safety policies could be generalized so they are applicable in future environmental crises. At the process level, ASD service agencies could set up client monitoring systems in order to catch if clients are experiencing disrupted access to ASD services. While we cannot change how the ASD care system reacted during COVID-19, we can learn from these events to increase its resilience and improve care for autistic children in the future.

## Footnotes

1 Both person-first (child with autism) and identity-first (autistic child) language are used by ASD researchers and stakeholders. We decided to use identity-first language for the current paper in order to reflect the perspectives of our autistic and neurodivergent colleagues.
